# Metabolomic Profiling and Antioxidant Capacity Changes in Longzi Black Barley During Germination

**DOI:** 10.3390/foods14122113

**Published:** 2025-06-16

**Authors:** Gan Hu, Yan Wei, Yuanhang Ren, Xinhui Wang, Dabing Xiang, Bin Li, Jinqiu Wang, Fang Geng

**Affiliations:** 1 Key Laboratory of Coarse Cereal Processing of Ministry of Agriculture and Rural Affairs, Sichuan Province Engineering Technology Research Center of Coarse Cereal Industrialization, Chengdu University, No. 2025 Chengluo Avenue, Chengdu 610106, China; hugan@cdu.edu.cn (G.H.); renyuanhang@cdu.edu.cn (Y.R.); wangxinhui@cdu.edu.cn (X.W.); gengfang@cdu.edu.cn (F.G.); 2 College of Food and Biological Engineering, Chengdu University, No. 2025 Chengluo Avenue, Chengdu 610106, China; 3 Chengdu National Agricultural Science and Technology Center, Chengdu University, No. 2025 Chengluo Avenue, Chengdu 610106, China; 4 College of Food Science and Technology, Huazhong Agricultural University, Wuhan 430070, China; libinfood@mail.hzau.edu.cn; 5 Key Laboratory of Environment Correlative Dietology, Ministry of Education, Huazhong Agricultural University, Wuhan 430070, China

**Keywords:** highland barley, germination, antioxidant capacity, metabolomic analysis, bioactive substances

## Abstract

Longzi black barley (*Hordeum vulgare* L. var. *nudum*, LBB), a highland barley variant with superior nutritional properties, has gained increasing attention for its health-promoting benefits. However, the metabolic changes during its germination process remain poorly understood. This study investigated the metabolic changes and antioxidant capacity during LBB germination. The results revealed significant dynamic changes in total flavonoid and total phenolic contents during germination, with the total flavonoids significantly decreasing by 32.59% initially (0–12 h, from 2.64 to 1.78 mg/g) and then slightly rebounding by 15.34% at 72 h, while the total phenolics decreased by 36.35% in the early stages (0–12 h, from 6.52 to 4.15 μmol/g) and increased markedly by 44.73% in the later stages (60–72 h, reaching 6.13 μmol/g) of germination. A metabolomic analysis identified 1015 metabolites, primarily including flavonoids, phenolic acids, amino acids and their derivatives, and alkaloids. During germination, the total flavonoid content continuously decreased by 24.24%, the phenolic acids showed no significant change, the amino acids and their derivatives increased significantly by 3.63-fold, and the alkaloid content increased slightly by 1.30-fold in the early stages (0–12 h) and significantly by 3.39-fold in the later stages (12–60 h). The study revealed the metabolic changes during the germination of LBB, providing scientific evidence for the further utilization of its nutritional value.

## 1. Introduction

Longzi black barley (*Hordeum vulgare* L. var. *nudum*, LBB) is a highland barley variant that is cold-resistant, drought-tolerant, highly adaptable, and has stable yields, even thriving at altitudes exceeding 4500 m. It serves as a staple resource on the Qinghai–Tibet Plateau in China [1]. Due to its rich nutritional content and unique functional properties, highland barley is regarded as a premium cereal crop. Compared to many other cereals, highland barley contains higher amounts of protein and unsaturated fats. Its starch content is around 60% to 75%, with dietary fiber ranging from 10.0% to 27.9%. The protein content ranges from 6.4% to 21%, including all eight essential amino acids, with lysine levels reaching up to 0.36%. Additionally, the fat content of highland barley is between 1.18% and 3.09%, primarily consisting of unsaturated fatty acids such as linoleic acid and oleic acid. Highland barley is also rich in β-glucan, γ-aminobutyric acid (GABA), and polyphenols, which contribute to its hypoglycemic and hypolipidemic properties [2,3]. The biochemical composition of highland barley is significantly influenced by geographical and environmental factors. Studies have shown that altitude significantly affects the content of bioactive compounds; highland barley growing at high altitudes (1200–3500 m) contains higher levels of β-glucan (7.5–30.8% higher), arabinoxylan (11.0–60.9% higher), and total anthocyanin (16.6–43.2% higher) than in flat areas. Temperature is reported as the main factor influencing lysine accumulation, while the sunshine duration and accumulated temperature are key factors affecting starch accumulation. For the protein content, the soil composition is the primary environmental factor, followed by climate conditions. Increases in monthly average temperature and sunshine hours promote protein accumulation, although simultaneous increases in altitude, monthly average temperature, and accumulated temperature may inhibit protein synthesis [4].

In recent years, as public health awareness has increased, germination has garnered attention as a method to significantly enhance the nutritional components and biological activities of plant seeds. Studies have shown that germination effectively increases the contents of dietary fiber, various essential amino acids, ω-6 and ω-3 fatty acids, vitamins, GABA, and other components in highland barley, while reducing the starch and fat contents [5,6]. Interestingly, highland barley is considered a low-glycemic index food, with predicted glycemic index values below 55 [7]. Consequently, highland barley is used to produce various products such as bread, noodles, cakes, wine, and vinegar [8].

Germination is a simple non-chemical method used to increase the bioactive compounds in grains. The research indicates that sprouted highland barley not only surpasses unsprouted barley in nutritional value but also excels in antioxidant, antibacterial, blood sugar regulation, and blood pressure regulation properties [9]. Zhu et al. evaluated the impact of germination on the functional components of black highland barley and found that the polyphenols and flavonoids in barley increased significantly by 2.8-fold and 3.9-fold, respectively, after germination [10]. Additionally, the contents of GABA and polyphenols in sprouted highland barley increased significantly, which are important for their roles in lowering blood sugar, reducing blood lipids, regulating the nervous system, and improving sleep, as well as anti-inflammation and anti-cancer activities [11,12]. Furthermore, sprouted highland barley can significantly improve the functional properties of food. Liu et al. demonstrated that sprouted highland barley significantly enhances the antioxidant capacity of fermented beverages, showing better market prospects [13]. Moreover, Liu et al. found that sprouted highland barley significantly improves the stability of dough, with notable increases in the dough’s nutritional content, water-holding capacity, water solubility index, and oil-holding capacity [14].

Despite the initial exploration of the impact of germination on the nutritional components and functional properties of highland barley, the research on the specific mechanisms and practical applications remains limited. Therefore, systematically understanding the chemical and biological changes during the germination process of LBB is crucial for developing high-quality, nutrient-rich, and functionally explicit sprouted highland barley products. This study aimed to investigate the changes in phenolic compounds and physiological activities during the germination of LBB and to further conduct a metabolomic analysis to explore the variations in small-molecule metabolites throughout the germination process. This involved in-depth research on relevant metabolic processes and an analysis of the metabolic differences in LBB before and after germination to systematically elucidate the characteristics of small-molecule metabolite changes during germination.

## 2. Materials and Methods

### 2.1. Materials and Reagents

The Longzi black barley (*Hordeum vulgare* L. var. *nudum*, LBB) seeds used in this study were sourced from the Tibet Autonomous Region. The LBB seeds were cleaned to remove impurities and stored at room temperature until further use. The total flavonoid content determination assay kit, the total phenolic content assay kit, the DPPH radical scavenging activity assay kit, the total antioxidant capacity (T-AOC) assay kit (ABTS method), and the total antioxidant capacity (T-AOC) assay kit (FRAP method) were purchased from Nanjing Jiancheng Bioengineering Institute (Beijing Solarbio Science and Technology Co., Ltd., Beijing, China). The analytical grade reagents were obtained from Sinopharm Chemical Reagent Co., Ltd. (Shanghai, China). All solutions were prepared using ultrapure water (Millipore, Billerica, MA, USA).

### 2.2. Germination Process

The LBB seeds were first disinfected via soaking in 0.1% sodium hypochlorite solution for 10 min, followed by thorough rinsing with distilled water. The seeds were then soaked in distilled water at a seed-to-water ratio of 1:5 (*w*/*v*) for 12 h at 23 °C. Untreated LBB seeds (LBB-0) served as the control. After soaking, the seeds were evenly spread on 9 cm petri dishes lined with two layers of filter paper, with about 30 seeds per dish. The dishes were then placed in a 23 °C incubator in the dark. The 72 h germination period was selected as our preliminary studies showed that LBB completes its germination phase during this period, transitioning from the late germination to seedling stage at 60–72 h. Throughout the culture period, the seeds were kept moist, ensuring the filter paper was damp without being submerged in water. Sampling was conducted every 12 h (LBB-12, LBB-24, LBB-36, LBB-48, LBB-60) until the end of the 72 h culture period (LBB-72). The harvested seeds from each time point were vacuum freeze-dried, ground using a mill, sieved through a 40-mesh screen, and stored at −20 °C for subsequent experiments.

### 2.3. Determination of Total Flavonoid Content

The total flavonoid content in the germinated barley samples was determined using a colorimetric method [15].

Total flavonoid extraction: The LBB samples were dried to a constant weight, ground, and passed through a 40-mesh sieve. Approximately 0.02 g of the dried and sieved sample was accurately weighed and 2 mL of extraction solvent was added. The mixture was shaken at 60 °C for 2 h for the extraction process. Following extraction, the mixture was centrifuged at 10,000 g at room temperature for 10 min. The supernatant was collected for a subsequent analysis.

Total flavonoid content determination: A 0.5 mL aliquot of the extract was mixed with 0.1 mL of 5% sodium nitrite solution. After 5 min, 0.1 mL of 10% aluminum chloride solution was added. After 5 min, 1 mL of 1 M sodium hydroxide solution was added, and the volume was adjusted to 5 mL with distilled water. After 15 min, the absorbance was measured at 502 nm using a UV–Vis spectrophotometer (UV-2600, Shimadzu, Kyoto, Japan). The total flavonoid content was expressed as mg of rutin equivalents per gram of dry weight (mg RE/g DW).

### 2.4. Determination of Total Phenol Content

The total phenol content in the germinated barley samples was determined using a spectrophotometry method [16].

Total phenol extraction: The LBB samples were dried to a constant weight, ground, and passed through a 40-mesh sieve. Approximately 0.1 g of the sieved powder was accurately weighed and 2 mL of 60% ethanol was added. Ultrasound-assisted extraction was performed with an ultrasonic power of 300 W, using a cycle of 5 s of sonication followed by 8 s of rest, at 60 °C for 30 min. The mixture was then centrifuged at 4000 rpm for 10 min, and the supernatant was collected for a subsequent analysis.

Total phenol content determination: A 0.5 mL aliquot of the extract was mixed with 2.5 mL of Folin–Ciocalteu reagent (diluted 1:10 with distilled water) and allowed to react for 5 min. Then, 2 mL of 7.5% sodium carbonate solution was added, and the mixture was incubated in the dark at room temperature for 1 h. The absorbance was measured at 760 nm using a UV–Vis spectrophotometer. The total phenolic content was expressed as μmol gallic acid equivalents/g (μmol/g).

### 2.5. Antioxidant Capacity Assays

Antioxidant capacity assays including DPPH radical scavenging activity, ABTS radical cation scavenging activity, and ferric-reducing antioxidant power (FRAP) assays were measured according to the previous method with some modifications [17].

#### 2.5.1. DPPH Radical Scavenging Activity

Sample pre-processing: Approximately 0.1 g of the LBB sample was accurately weighed and finely chopped. The sample was then homogenized in an ice water bath with 1 mL of 80% methanol solution. The homogenate was centrifuged at 12,000 rpm for 10 min. The supernatant was collected and kept on ice for a subsequent analysis.

Antioxidant capacity determination: A 0.1 mL aliquot of the supernatant was mixed with 3.9 mL of 0.1 mM DPPH solution in methanol. The mixture was shaken and incubated in the dark at room temperature for 30 min. The absorbance was measured at 517 nm using a UV–Vis spectrophotometer. The DPPH radical scavenging activity was calculated as a percentage of inhibition using the following formula:

(1) $$\begin{array}{c} \mathrm{DPPH}\;\left(µg\;\mathrm{Trolox}/g\right)=\mathrm{Concentration}\;\mathrm{of}\;\mathrm{Trolox}\;\left(µg/mL\right)\times \\ \frac{\mathrm{Volume}\;\mathrm{of}\;\mathrm{extraction}\;\mathrm{solvent}\left(mL\right)}{\mathrm{weight}\;\mathrm{of}\;\mathrm{sample}\left(g\right)}\; \end{array}$$

#### 2.5.2. ABTS Radical Cation Scavenging Activity

Sample pre-processing: The LBB sample was mixed with physiological saline at a weight (g)-to-volume (mL) ratio of 1:9. The mixture was homogenized mechanically in an ice water bath to ensure thorough cell disruption and the release of antioxidants. The homogenate was then centrifuged at 120 rpm for 5 min at 4 °C. The supernatant was collected for subsequent antioxidant measurements.

Antioxidant capacity determination: ABTS radical cations were generated by mixing 7 mM of ABTS solution with 2.45 mM of potassium persulfate and allowing the mixture to stand in the dark at room temperature for 12–16 h. A 0.1 mL aliquot of the supernatant was mixed with 3.9 mL of the diluted ABTS solution. The mixture was incubated at room temperature for 6 min, and the absorbance was measured at 734 nm. The ABTS radical cation scavenging activity value was expressed as mM of Trolox equivalents per gram of dry weight (mM TE/g DW).

#### 2.5.3. Ferric-Reducing Antioxidant Power (FRAP) Assay

Sample pre-processing: The LBB sample was mixed with physiological saline at a weight (g)-to-volume (mL) ratio of 1:4. The mixture was mechanically homogenized in an ice water bath to ensure thorough cell disruption and the release of antioxidants. The homogenate was then centrifuged at 120 rpm for 5 min at 4 °C. The supernatant was collected for subsequent antioxidant measurements.

Antioxidant capacity determination: The FRAP reagent was prepared by mixing 300 mM of acetate buffer (pH 3.6), 10 mM of TPTZ solution in 40 mM HCl, and 20 mM of FeCl_3_·6H_2_O solution in a ratio of 10:1:1 (*v*/*v*/*v*). A 0.1 mL aliquot of the supernatant was mixed with 3 mL of the FRAP reagent and incubated at 37 °C for 30 min. The absorbance was measured at 593 nm. The FRAP value was expressed as mM of Trolox equivalents per gram of dry weight (mM TE/g DW).

### 2.6. Metabolomic Analysis

A metabolomic analysis of the germinated barley samples was carried out according to the previous method with some modifications [18].

#### 2.6.1. Sample Preparation

Based on the germination stages, LBB samples were selected for the metabolomic analysis at 0 h (LBB-0), 12 h (LBB-12), and 60 h (LBB-60) of germination. These time points were selected based on our previous research identifying key developmental stages; 12 h represents the early germination stage with initial water absorption and metabolic activation, while 60 h marks the late germination stage when the coleoptile breaks through the grain husk. The 72 h point was not included as it represents the completed seedling stage rather than the germination process. The samples were quickly chopped and ground into a fine powder in liquid nitrogen. Three biological replicates were prepared for each germination stage for metabolomic sequencing.

#### 2.6.2. Metabolite Extraction Procedure

The samples were vacuum freeze-dried and ground into a fine powder. Approximately 100 mg of the powdered sample was dissolved in 1.2 mL of extraction solvent (70% methanol). The mixture was vortexed for 30 s every 30 min, with this process repeated six times. The processed samples were then stored overnight at 4 °C. After standing, the samples were centrifuged and the supernatant was filtered through a 0.22 μm microporous membrane for the UPLC-MS/MS analysis.

#### 2.6.3. Chromatography and Mass Spectrometry Data Acquisition

The data acquisition was performed using ultra-performance liquid chromatography (UPLC) and tandem mass spectrometry (MS/MS). The chromatography column conditions followed the previous method [19] with some modifications; the injection volume was 4 μL, with a flow rate of 0.35 mL/min and a column temperature of 40 °C. The gradient elution conditions are shown in Table S1, and the mass spectrometry conditions are listed in Table S2.

#### 2.6.4. Differential Metabolite Analysis

The obtained mass spectrometry data were compared with public metabolite databases to identify metabolites. Differential abundance metabolites were screened based on fold changes (FC ≥ 2 or FC ≤ 0.5) and the variable importance in projection (VIP ≥ 1). Metabolites in the samples were identified based on the home-made MWDB (metadata database) and other databases, and a qualitative analysis of the information and secondary general data was carried out according to *m*/*z*. The metabolite structure analysis referred to some existing mass spectrometry public databases, mainly including massbank (https://www.massbank.jp/ accessed on 6 July 2024), HMDB (https://www.hmdb.ca/ accessed on 6 July 2024), Metlin (https://metlin.scripps.edu/index.php accessed on 6 July 2024), and Mona (https://mona.fiehnlab.ucdavis.edu/ accessed on 6 July 2024).

### 2.7. Statistical Analysis

The experimental data were expressed by the mean ± standard deviation values of three sets of replicates, the analyzed and visualized using IBM SPSS 20.0 (IBM, Chicago, IL, USA) and GraphPad Prism v9 (GraphPad, San Diego, CA, USA). A one-way analysis of variance (ANOVA) followed by a Tukey post hoc test was performed to determine statistical comparisons, and a value of *p* < 0.05 was considered statistically significant.

## 3. Results

### 3.1. Determination of Bioactive Substances and Antioxidant Capacity

**Figure 1:**
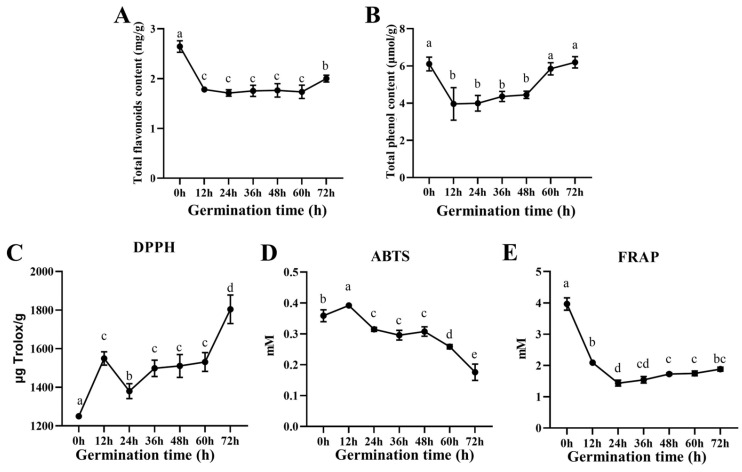
Changes in physiological active substances and antioxidant capacity: (**A**) total flavonoid content; (**B**) total phenol content; (**C**) DPPH radical scavenging capacity; (**D**) ABTS radical scavenging activity; (**E**) FRAP assay. Different letters indicate significant differences between time points (*p* < 0.05). Data are presented as the mean ± SD (*n* = 3).

Dynamic changes in bioactive compounds were observed throughout the germination process of the LBB. As shown in Figure 1A, the initial total flavonoid content at 0 h was 2.64 mg/g, which decreased markedly by 32.59% during the first 12 h of germination. This significant decrease can be attributed to several factors, including the leaching of water-soluble flavonoids during the soaking process, the rapid metabolism of stored flavonoids to provide energy for embryonic tissue activation, and the enzymatic breakdown of flavonoid glycosides by activated glycosidases upon imbibition [20]. This was followed by a relatively stable period between 12 and 60 h, before showing a modest increase of 15.34% at 72 h compared to 60 h. The final total flavonoid content at 72 h was 2.00 mg/g, representing a 24.24% reduction from the initial value.

Similarly, the total phenolic content exhibited distinct phase-dependent changes. Starting at 6.52 μmol/g in LBB-0, it decreased significantly to 4.15 μmol/g at 12 h. The content remained relatively constant during the mid-germination period (12–48 h) before showing a substantial increase during seedling formation (60–72 h), ultimately reaching 6.13 μmol/g (Figure 1B). These patterns suggest an initial mobilization of stored compounds followed by de novo synthesis during later developmental stages [21].

The antioxidant capacity of the LBB at different germination stages was evaluated using DPPH, ABTS, and FRAP assays. The DPPH radical scavenging activity showed a complex pattern, with a significant initial increase (23.98%) in early germination, followed by a 10.64% decrease within the first 12 h of mid-germination, then a 7.79% increase between 24–36 h, and finally a gradual rise in later stages. By 72 h, the DPPH radical scavenging activity reached 1803.697 ± 65.959 µg Trolox/g, representing a 1.44-fold increase compared to 0 h (Figure 1C). In contrast, the ABTS radical scavenging activity showed a progressive decline throughout germination, with the most pronounced decrease occurring between 48 and 72 h (Figure 1D). The FRAP assay revealed a dramatic initial decrease in ferric-reducing antioxidant power, dropping by 2.8-fold within the first 24 h, followed by a modest but consistent increase from 24 to 72 h, ultimately reaching 47.46% of the initial value (Figure 1E).

These divergent trends among the antioxidant assays reflect the complex dynamics of different antioxidant compounds during germination. The increase in DPPH scavenging activity correlates with the accumulation of amino acids and alkaloids, particularly during later stages, as these compounds can act as hydrogen donors. The decline in ABTS values, especially pronounced between 48–72 h, aligns with the decrease in total flavonoids, suggesting that flavonoids are major contributors to ABTS radical scavenging. The dramatic decrease in FRAP values during early germination (0–24 h) further supports this correlation, as flavonoids are known to be potent reducing agents [22]. The different values obtained from the DPPH, ABTS, and FRAP assays can be attributed to their distinct reaction mechanisms and specificities. DPPH mainly measures the hydrogen-donating ability of antioxidants, ABTS evaluates both hydrophilic and lipophilic antioxidants, while FRAP specifically measures the reducing power of antioxidants. These complementary methods provide a comprehensive assessment of the antioxidant capacity [23]. The observed dynamic changes in antioxidant activities reflect the metabolic adjustments during germination. The initial decrease in antioxidant capacity corresponds to the mobilization of stored compounds for early growth, while the later stage changes indicate the activation of biosynthetic pathways producing new antioxidants [24].

### 3.2. Metabolomic Analysis

#### 3.2.1. Identification and Quantification of Metabolites

**Figure 2:**
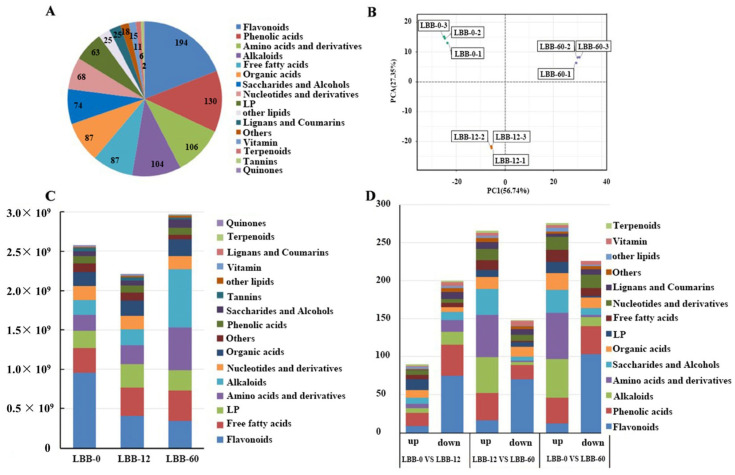
Metabolomic profiling of LBB during germination: (**A**) distribution of identified metabolites by chemical classification; (**B**) A principal component analysis (PCA) score plot of metabolites in LBB samples at different germination stages (LBB-0, LBB-12, and LBB-60, with three biological replicates each); (**C**) changes in total abundance of different metabolite classes during germination; (**D**) numbers of up- and down-regulated metabolites in pairwise comparisons between germination stages (fold change ≥2 or ≤0.5, VIP ≥ 1).

A quantitative metabolomic analysis was performed on LBB-0, LBB-12, and LBB-60, focusing on the changes in metabolites during the initiation of germination (LBB-12/LBB-0) and mid-to-late stages of germination (LBB-60/LBB-12). Through the LC-MS/MS analysis, a total of 1015 metabolites were identified (Figure 2A). These metabolites were classified into major categories, with flavonoids being the most abundant (194, 19.11%), followed by phenolic acids (130, 12.81%), amino acids and their derivatives (106, 10.44%), alkaloids (104, 10.25%), organic acids (87, 8.57%), free fatty acids (87, 8.57%), sugars and alcohols (74, 7.29%), nucleotides and their derivatives (68, 6.70%), and lysophospholipids (63, 6.21%). Notably, flavonoids and alkaloids emerged as the predominant secondary metabolites.

A principal component analysis (PCA) was employed to evaluate the metabolic distinctions among the samples at different germination stages. The PCA score plot demonstrated clear clustering of biological replicates within each group while showing distinct separation between groups (Figure 2B), indicating good intragroup reproducibility and significant intergroup differences. The first principal component (PC1) and the second principal component (PC2) explained 56.74% and 27.35% of the variance, respectively. On PC1, the samples from the three stages separated from each other with increasing germination time. This clear separation suggested that each germination stage was characterized by a unique metabolic profile, reflecting the specific biochemical requirements and activities of the seed at each stage of development [25].

The quantitative analysis of the relative metabolite abundance based on the mass spectrometry intensity revealed stage-specific metabolic profiles (Figure 2C). In ungerminated seeds (LBB-0), the five most abundant metabolite categories were flavonoids, free fatty acids, lysophospholipids, amino acids and their derivatives, and alkaloids. During germination, distinct temporal patterns emerged; flavonoids showed a progressive decline, free fatty acids and amino acids and their derivatives demonstrated continuous increases, while alkaloids exhibited a biphasic response with modest early-stage increases (0–12 h) followed by more substantial accumulation in later stages. Lysophospholipids displayed unique dynamics, increasing during early germination before declining in the mid-to-late stages.

The screening for differential metabolites revealed 290 differential metabolites between LBB-0 and LBB-12 (99 upregulated, 191 downregulated), with flavonoids (9 upregulated, 75 downregulated), phenolic acids (17 upregulated, 41 downregulated), and alkaloids (6 upregulated, 17 downregulated) being the most numerous categories. Between LBB-12 and LBB-60, 414 differential metabolites were identified (266 upregulated, 148 downregulated), with flavonoids (16 upregulated, 70 downregulated), amino acids and their derivatives (56 upregulated, 2 downregulated), and phenolic acids (36 upregulated, 19 downregulated) being the predominant categories. Between LBB-0 and LBB-60, 504 differential metabolites were identified (276 upregulated, 228 downregulated), including 115 flavonoids, 71 phenolic acids, and 64 amino acids and their derivatives. Across the three stages, 93 common differential metabolites were identified, with flavonoids, phenolic acids, alkaloids, and amino acids and their derivatives being the primary categories showing significant changes during germination. The progressive decline in flavonoids during germination reveals a complex metabolic transition in developing seeds. The rapid initial decrease likely represents more than simple degradation; it suggests a programmed mobilization of these compounds to serve multiple physiological functions. As strong electron donors, flavonoids may act as crucial redox regulators during the transition from dormancy to active metabolism [26]. Their depletion correlates with increased respiratory activity and cellular differentiation, suggesting their involvement in managing the oxidative burst that typically accompanies early germination events. The sustained decrease through mid-germination indicates their continuing role in developmental processes, possibly serving as precursors for cell wall components or as signaling molecules mediating growth responses. The selective degradation of specific flavonoids, particularly anthocyanins, suggests a precisely controlled metabolic hierarchy rather than random utilization, reflecting the evolutionary optimization of seed germination processes [27]. The increase in amino acids and their derivatives indicated enhanced protein degradation and amino acid synthesis, which were critical for providing the necessary building blocks for new protein synthesis during seedling development [28]. The observed increase in alkaloids suggested an upregulation of secondary metabolite biosynthesis pathways, which may play a role in the plant’s defense mechanisms and stress responses during germination. Alkaloids are known for their diverse biological activities, including antimicrobial and insecticidal properties, which could be beneficial for the emerging seedlings [29].

#### 3.2.2. Phenolic Metabolites and Metabolic Pathways

Highland barley is rich in phenolic compounds, including phenolic acids and flavonoids, which possess strong antioxidant, antiproliferative, and cholesterol-lowering activities [30]. The increase in phenolic compounds during seed germination may be due to de novo synthesis and transformation, as well as the degradation of cell wall enzymes, which releases phenolic compounds and significantly increases their content. However, some phenolic compounds become undetectable after germination, likely due to partial oxidation and degradation during the process [21,31].

(1) Flavonoid metabolites and metabolic pathways

**Figure 3:**
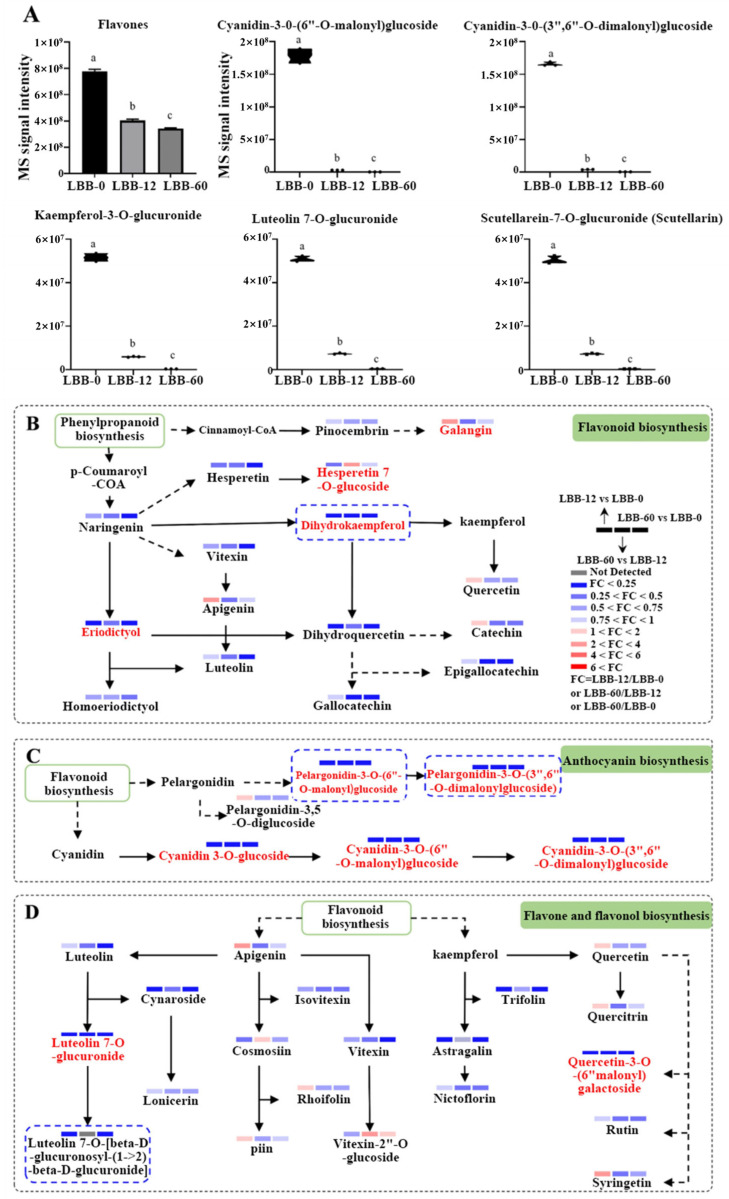
An analysis of flavonoid metabolites during LBB germination: (**A**) changes in abundance of major flavonoid compounds during germination (different letters indicate significant differences at *p* < 0.05); (**B**) metabolic pathway of flavonoid biosynthesis; (**C**) metabolic pathway of anthocyanin biosynthesis; (**D**) metabolic pathway of flavone and flavonol biosynthesis. The colored bars indicate fold changes (FC) between different germination stages (LBB-12 vs. LBB-0, LBB-60 vs. LBB-0, and LBB-60 vs. LBB-12).

The metabolomic data revealed that the total flavonoid abundance in LBB-0 decreased by 48.07% in LBB-12 and remained relatively low throughout the subsequent germination stages. The top five flavonoids in LBB-0—cyanidin-3-O-(6″-O-malonyl)glucoside, cyanidin-3-O-(3″,6″-O-dimalonyl)glucoside, kaempferol-3-O-glucuronide, luteolin-7-O-glucuronide, and scutellarin—showed a similar trend, with their abundance decreasing during germination (Figure 3A), which aligns with the observed changes in total flavonoid content (Figure 1A).

The analysis of 39 flavonoid metabolites mapped to KEGG pathways showed significant changes in their abundance (Figure 3B–D). Among the detected flavonoids, 30 exhibited significant changes during germination (Table S3). In the metabolic pathway map, 11 metabolites were highlighted; galangin increased during early germination but decreased in the later stages, while naringenin-7-O-glucoside decreased during early germination and increased in the later stages. The remaining nine—including dihydrokaempferol (naringenin), eriodictyol, pelargonidin-3-O-(6″-O-malonyl)glucoside, pelargonidin-3-O-(3″,6″-O-dimalonyl)glucoside, cyanidin-3-O-glucoside, cyanidin-3-O-(6″-O-malonyl)glucoside, cyanidin-3-O-(3″,6″-O-dimalonyl)glucoside, luteolin-7-O-glucuronide, and quercetin-3-O-(6″-malonyl)galactoside—decreased throughout germination. Notably, four of these compounds (dihydrokaempferol, pelargonidin-3-O-(6″-O-malonyl)glucoside, pelargonidin-3-O-(3″,6″-O-dimalonyl)glucoside, and luteolin-7-O-glucuronide) were undetectable in LBB-60, indicating complete degradation during germination, with two found in the anthocyanin biosynthesis pathway. Overall, the flavonoid abundance primarily decreased, particularly for anthocyanins.

The overall decline in flavonoid metabolites, as indicated by both the total flavonoid content and metabolomic data, can be attributed to three main factors during early germination (0–12 h), namely the leaching of water-soluble flavonoids during imbibition, the metabolization of stored flavonoids for providing energy, and the enzymatic breakdown of flavonoid glycosides for early seedling development. This decline leads to reduced antioxidant capacity and affects the nutritional properties of the germinated LBB. Specifically, the decrease in anthocyanins reflects the reduced need for UV protection and seed coat integrity as the seedling establishes its own defensive mechanisms during development [32].

Other pathways also showed a general decrease, although some compounds, such as galangin, apigenin, quercetin, catechin, pelargonidin-3,5-O-diglucoside, quercitrin, myricitrin, apigetrin, and eupatorin, increased during early germination, while naringenin-7-O-glucoside, cosmosiin, and vitexin-2″-O-glucoside increased in the later stages.

(2) Phenolic Acid Metabolites and Metabolic Pathways

**Figure 4:**
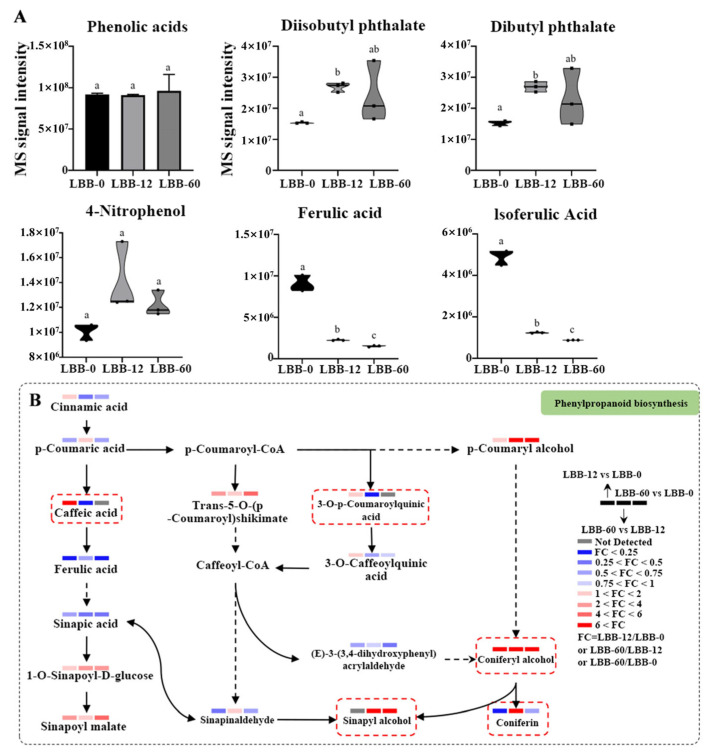
An analysis of phenolic acid metabolites during LBB germination: (**A**) changes in abundance of major phenolic acid compounds during germination (different letters indicate significant differences at *p* < 0.05); (**B**) metabolic pathway of phenylpropanoid biosynthesis. The colored bars indicate fold changes (FC) between different germination stages (LBB-12 vs. LBB-0, LBB-60 vs. LBB-0, and LBB-60 vs. LBB-12). Compounds in red dashed boxes represent key intermediates in the pathway.

In LBB-0, the top three phenolic acids—dibutyl phthalate, 4-nitrophenol, diisobutyl phthalate—showed a slight increase in LBB-12 but decreased in LBB-60, ultimately increasing compared to LBB-0. Ferulic acid consistently decreased during germination, while isoferulic acid decreased in LBB-12 and increased in LBB-60. The trend of phenolic acid metabolite abundance mirrored that of dibutyl phthalate and other similar metabolites, although the overall phenolic acid abundance did not significantly change across the three stages (Figure 4A).

The metabolomic data analysis revealed that the phenolic acid metabolites were distributed in the “phenylpropanoid biosynthesis” pathway and its upstream and downstream pathways. Sixteen phenolic acid metabolites identified in the KEGG pathway were mapped to analyze changes in abundance (Figure 4B). Eighteen differential phenolic acid metabolites showed significant changes during germination (Table S3). Five compounds—p-coumaroyl alcohol, trans-5-O-p-coumaroylshikimic acid, 1-O-sinapoyl-D-glucose, sinapoylmalate, and coniferyl alcohol—increased throughout germination, while three—ferulic acid, sinapinic acid, and caffealdehyde—decreased.

The changes in caffeic acid, 3-O-p-coumaroylquinic acid, coniferyl alcohol, coniferin, and sinapyl alcohol were more pronounced. Caffeic acid and 3-O-p-coumaroylquinic acid were undetectable in LBB-0 and LBB-60 but increased in LBB-12. Coniferyl alcohol was undetectable in ungerminated seeds but increased during germination and was detected in LBB-12, continuing to increase in the later stages. Coniferin was detectable in LBB-0 and LBB-60 but not in LBB-12. Sinapyl alcohol was synthesized and significantly increased in the mid-to-late germination stages, being detectable in LBB-60.

These metabolic profiles reveal a precisely regulated phenolic acid network during germination. The temporal changes of specific compounds—such as the gradual increase in p-coumaroyl alcohol and sinapoylmalate, coupled with the decrease in ferulic and sinapinic acids—suggest targeted metabolic redirection supporting seedling development [33]. The stage-specific emergence of compounds (e.g., sinapyl alcohol in LBB-60) and transient appearance of others (caffeic acid in LBB-12) indicate their roles as key intermediates in lignin biosynthesis. While the total phenolic acid content remained relatively stable, these coordinated shifts in individual compounds, particularly within the phenylpropanoid pathway, demonstrate the strategic allocation of phenolic resources between structural development and defense during germination [34,35].

#### 3.2.3. Amino Acids and Their Derivatives

**Figure 5:**
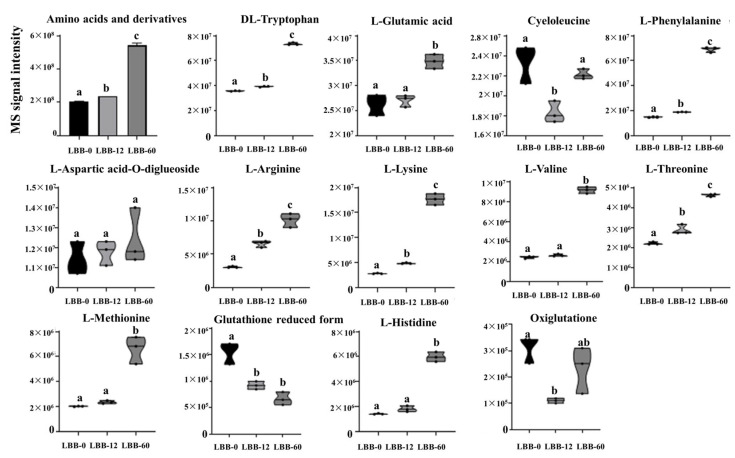
Changes in abundance of amino acids and their derivatives during LBB germination. Different letters above the bars or violin plots indicate significant differences between germination stages (*p* < 0.05). The MS signal intensity reflects the relative abundance of each compound at different germination time points (LBB-0, LBB-12, and LBB-60). Violin plots show the distribution of biological replicates.

The metabolomic analysis revealed that amino acids and their derivatives were the third most abundant category of metabolites in the germinating LBB, comprising 106 compounds (10.44% of the total). The total abundance of amino acids and their derivatives significantly increased throughout the germination process, with a 3.63-fold increase at 60 h compared to 12 h. In LBB-0, the top five amino acids and their derivatives by abundance were cycloleucine, DL-tryptophan, L-glutamic acid, L-phenylalanine, and L-aspartic acid-O-diglucoside, all of which followed the general increasing trend (Figure 5).

Five essential amino acids (L-lysine, L-phenylalanine, L-methionine, L-threonine, and L-valine) and two semi-essential amino acids (L-arginine and L-histidine) were detected in samples from all three stages. The relative abundance levels of these amino acids varied significantly across different germination stages. L-lysine, L-methionine, L-threonine, L-valine, and L-arginine showed significant increases in both the early and late germination stages, while L-phenylalanine and L-histidine exhibited no significant changes in the early stages but increased significantly in the later stages (Figure 5). The total content of essential amino acids increased by 1.30-fold in the early germination stages and by 3.39-fold in the later stages, while the total content of semi-essential amino acids increased by 1.87-fold in the early stages and by 1.92-fold in the later stages. The observed increase in essential and semi-essential amino acids highlights the enhanced metabolic activity required to support seedling growth and development [36]. During the germination of LBB, the total free amino acid content continuously increased, with essential amino acids increasing by 5.76-fold and non-essential amino acids by 1.64-fold at 72 h.

The accumulation of amino acids during germination indicates a metabolic shift from protein degradation to active biosynthesis, preparing for seedling establishment. The preferential increase in essential amino acids suggests targeted metabolic regulation rather than random protein breakdown [28]. The parallel changes in the glutathione system, marked by an initial GSH decrease and later GSSG stabilization, reveal an integrated redox signaling network orchestrating this metabolic transition. This coordinated response likely facilitates the switch from dormancy to active growth through thiol-based regulation [37].

#### 3.2.4. Alkaloid Metabolites

Alkaloids, known for their broad biological activity, are a prominent class of nitrogen-containing organic compounds predominantly derived from plants [38]. In the germinating LBB, 104 alkaloid metabolites were identified, accounting for 10.25% of the total metabolites. The overall abundance of alkaloids increased slightly in the early germination stages but rose significantly during the mid-to-late stages.

**Figure 6:**
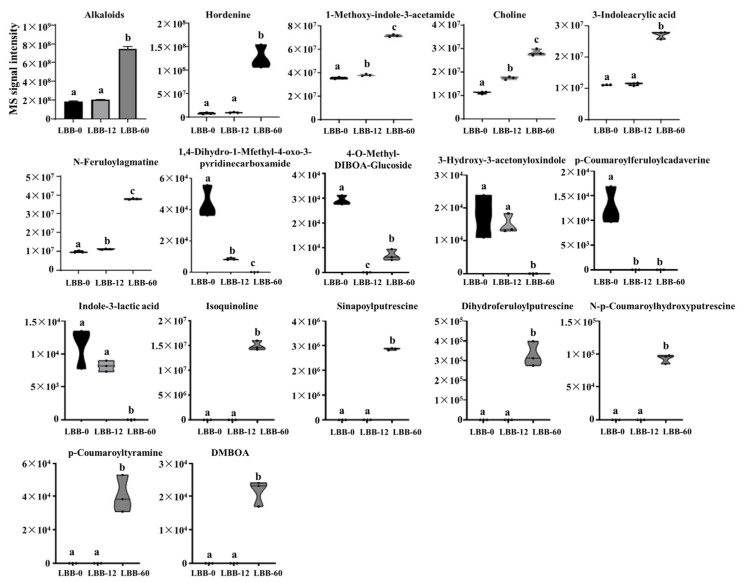
Changes in abundance of alkaloid metabolites during LBB germination. The relative abundance of total alkaloids and individual alkaloid compounds is shown as the MS signal intensity at different germination stages (LBB-0, LBB-12, and LBB-60). Different letters indicate significant differences between germination stages (*p* < 0.05). The violin plots represent the distribution of biological replicates for each compound.

The major types of alkaloids detected include phenolamines (36), alkaloids (32), indole alkaloids (21), piperidine alkaloids (4), pyridine alkaloids (4), quinoline alkaloids (3), pyrrole alkaloids (3), and isoquinoline alkaloids (1). In LBB-0, the top five alkaloids by abundance were hordenine, 3-indoleacrylic acid, choline, N-feruloylputrescine, and 1-methoxyindole-3-acetamide, all of which showed trends consistent with the overall pattern (Figure 6).

Notably, six alkaloids were not detected in LBB-0 and LBB-12 but showed significant increases in LBB-60, including isoquinoline, sinapoylspermidine, dihydrosinapoylspermidine, N-p-coumaroyl hydroxyputrescine, p-coumaroyltyramine, and 6,7-dimethoxy-2-benzoxazolinone. Conversely, three compounds—3-hydroxy-3-acetylmethylindoline, 1,4-dihydro-1-methyl-4-oxopyridine-3-carboxamide, and indole-3-lactic acid—were detected in LBB-0 and LBB-12 but not in LBB-60. N-feruloyltyramine was present in LBB-0 but decreased to undetectable levels in LBB-12 and LBB-60. Meanwhile, 4-O-methyl-DIBOA-glucoside decreased in LBB-12 and was undetectable but present in LBB-0 and LBB-60. The alkaloid accumulation pattern during germination reveals distinct functional phases; an initial modest increase supports germination signaling, while later substantial accumulation enhances seedling defense [39]. The preferential synthesis of phenolamines and indole alkaloids suggests their targeted roles in stress response and cellular differentiation. This metabolic investment in alkaloids indicates their importance in both the seedling development and nutritional enhancement of germinated LBB.

## 4. Conclusions

A comprehensive metabolomic analysis of LBB germination revealed significant dynamic changes in bioactive compounds and metabolic profiles. During germination, the total flavonoid content significantly decreased initially (0–12 h) and slightly rebounded later, while the total phenolics increased markedly in the later stages (60–72 h). Antioxidant capacity measurements showed significant fluctuations in DPPH free radical scavenging ability, while the ABTS and FRAP antioxidant capacities demonstrated a declining trend. A metabolomic analysis identified 1015 metabolites, with 551 showing significant differential expression across germination stages. Among the major metabolite categories, the flavonoids showed a continuous decline throughout germination, while the phenolic acids maintained relatively stable levels. The amino acids and their derivatives increased significantly during germination, particularly in the later stages. The alkaloids exhibited a biphasic response with modest increases during early germination (0–12 h) followed by substantial accumulation in the later stages (12–60 h). While this study provides crucial insights into metabolic reprogramming during LBB germination, limitations including the discrete sampling points and unexplored environmental influences should be considered. Future research studies should focus on more frequent sampling intervals and environmental factor effects. Nevertheless, these findings offer valuable guidance for optimizing germination conditions and developing functional food products with enhanced nutritional properties.

## Data Availability

The original contributions presented in the study are included in the article/Supplementary Materials. Further inquiries can be directed to the corresponding author.

## Supplementary Materials

The following supporting information can be downloaded at https://www.mdpi.com/article/10.3390/foods14122113/s1: Table S1. Mobile-phase elution gradient. Table S2. Mass spectrometry parameters. Table S3 Differential metabolites between groups.
