# Supplementary material for: Metabolomic Profiling and Antioxidant Capacity Changes in Longzi Black Barley During Germination
Source: Foods. 2025 Jun 16;14(12):2113. doi: 10.3390/foods14122113 (PMC12191945; doi:10.3390/foods14122113)
Supplement: Supplementary file 1 [file foods-14-02113-s001.zip › foods-3666153-supplementary.pdf]

foods

MDPI

Metabolomic Profiling and Antioxidant Capacity Changes in Longzi Black Barley During Germination

Supplementary Materials: The following supporting information can be downloaded at: https://www.mdpi.com/article/doi/s1, Table S1: Mobile-phase elution gradient; Table S2: Mass spectrometry parameters.

Table S1 Mobile-phase elution gradient (Phase A: 0.1% formic acid in water; Phase B: 0.1% formic acid in acetonitrile).

Time（min）	Flow rate（mL/min）	Phase A（%）	Phase B（%）
0	0.35	95	5
1	0.35	85	15
2	0.35	75	25
3	0.35	65	35
4	0.35	55	45
5	0.35	45	55
6	0.35	35	65
7	0.35	25	75
8	0.35	15	85
9	0.35	5	95
10	0.35	5	95
11.10	0.35	95	5
14	0.35	95	5

Table S2. Mass spectrometry parameters.

Description	Parameter
Ion source	Turbospray
Source temperature (℃)	550
Sheath gas flow rate (arb)	50
Ion spray voltage (V)	5500 (Positive mode), -4500 (Negative mode)
Ion source gas I (psi)	50
Ion source gas II (psi)	60
Curtain gas(psi)	25.0
QQQ scan	MRM Mode
Collision gas	Medium

Table S3. Differential metabolites for two-by-two comparisons between groups

Note: This table shows the change in abundance of the same differential metabolites that changed significantly at all three germination stages, FC is the ratio of abundance between two by two, and all DAMs p-value< 0.01.

ID	Metabolite	Classification	$FC_{(LBB-12/LBB-0)}$	$FC_{(LBB-60/LBB-12)}$
		**LBB-12/LBB-0 up and LBB-60/LBB-12 up**		
mws0609	Guanosine 3',5'-cyclic mono-phosphate	Nucleotides and de-rivatives	12144.407	3.084
mws2125	Phosphoenolpyruvate	Organic acids	9970.852	2.588
Lmsn002247	1-O-Salicyloyl-β-D-glucose*	Phenolic acids	4178.296	2.924
Zmhn001358	4-O-Glucosyl-4-hydroxyben-zoic acid*	Phenolic acids	2748.185	3.293
pme1419	L-Methionine methyl ester	Amino acids and de-rivatives	2039.963	4.722
pmb2922	Uridine 5'-diphospho-D-glu-cose	Nucleotides and de-rivatives	35.314	10.286
Zmfn000481	Uridine-5'-Diphosphate-D-Xylose	Nucleotides and de-rivatives	31.018	5.332
mws0866	D-Glucose 6-phosphate	Saccharides and Al-cohols	28.074	8.172
pme3313	D-Fructose 6-phosphate	Saccharides and Al-cohols	15.041	4.375
Zmhn000892	4-O-Glucosyl-3,4-dihy-droxybenzyl alcohol	Phenolic acids	4.108	11.966
Lmbp000123	L-Homomethionine	Amino acids and de-rivatives	3.242	4.948
Hmqn000843	Tachioside	Phenolic acids	2.843	10.506
MWS4296	Glycylphenylalanine	Amino acids and de-rivatives	2.771	5.031
Lmhp001670	L-Valyl-L-Leucine	Amino acids and de-rivatives	2.626	6.904
MWS4309	Glycyl-tryptophan	Amino acids and de-rivatives	2.452	4.228
Lmhp001461	L-Prolyl-L-Leucine	Amino acids and de-rivatives	2.383	8.414
Lmhp005550	N,N'-Diferuloylputrescine	Alkaloids	2.376	11.808
pmb2654	Anthranilate-1-O-Sophoroside	Phenolic acids	2.297	2.553
Lmhp002001	L-Valyl-L-Phenylalanine	Amino acids and de-rivatives	2.260	9.431
Lmhp007836	LysoPE 16:3	LPE	2.185	3.152
Lmhp002031	L-Leucyl-L-Leucine	Amino acids and de-rivatives	2.130	14.459

mws5041	L-Glycyl-L-isoleucine	Amino acids and derivatives	2.106	5.010
**LBB-12/LBB-0 up and LBB-60/LBB-12 down**				
pmc0960	LysoPC 20:4	LPC	13725.556	0.00007
Lmhp009368	LysoPC 22:6	LPC	9316.704	0.0001
Lmhp008742	LysoPC 20:5	LPC	1978.296	0.001
pmn001421	3-O-p-Coumaroylquinic acid	Phenolic acids	1715.444	0.001
pmf0096	Oxalic acid	Organic acids	12.663	0.270
pma1751	N-(beta-D-Glucosyl)nicotinate	Others	10.010	0.285
pmp001236	Clove chromone	Others	5.121	0.00007
mws4173	5,7-Dimethoxycoumarin (Limettin)(Citropten)	Lignans and Coumarins	4.861	0.026
MWSmce539	Dibutyl sebacate	Free fatty acids	3.560	0.018
mws0036	Hesperetin-7-O-rutinoside (Hesperidin)	Flavonoids	2.859	0.290
pmb2165	LysoPC 10:0	LPC	2.265	0.422
Lmyn006227	Galangin (3,5,7-Trihydroxyflavone)	Flavonoids	2.265	0.373
pme3011	γ-Aminobutyric acid	Organic acids	2.122	0.434
LBB-12/LBB-0 down and LBB-60/LBB-12 up				
Lmhn003240	Benzoylmalic acid	Phenolic acids	0.492	170.909
Zmjp003291	"""Vitexin-2""""-O-galactoside"""	Flavonoids	0.489	4.091
mws1375	Nicotianamine	Alkaloids	0.442	3.193
Smcp000882	N-benzoyl-2-aminoethyl-β-D-glucopyranoside	Others	0.438	10.509
pmp000579	Diosmetin-7-O-galactoside*	Flavonoids	0.438	2.031
pmb0498	Sinapoylagmatine	Alkaloids	0.425	237.250
Hmmp004965	Diosmetin-7-O-glucoside*	Flavonoids	0.416	2.121
Hmgp002189	Hispidulin-7-O-Glucoside	Flavonoids	0.414	2.118
Hmmp003156	Saponarin(Isovitexin-7-O-glucoside)	Flavonoids	0.396	4.271
Lmhp002501	N-Feruloylhydroxyputrescine	Alkaloids	0.391	17.109
Lmzp002365	Hesperetin-7-O-glucoside	Flavonoids	0.350	2.397
mws0344	D-Xylonic acid	Saccharides and Alcohols	0.338	2.065
NK10253223	2-Amino-3-methoxybenzoic acid	Phenolic acids	0.338	3.253
Hmgn004693	8-Hydroxy-α-conidendrin	Lignans and Coumarins	0.310	2.798
Hmgn002833	4-Ketopinoresinol	Others	0.254	3.315
Rfmb26201	Syringaresinol-4'-O-(6''-acetyl)glucoside	Lignans and Coumarins	0.231	2.313
Zmhn001793	6-O-Caffeoyl-D-glucose	Phenolic acids	0.210	3.023

MWS20152	Syringaresinol	Lignans and Coumarins	0.195	2.091
Zmgn000173	D-Ribose	Saccharides and Alcohols	0.194	3.792
pmb2871	1-O-Gentisoyl-D-glucoside	Phenolic acids	0.179	4.272
Zmhn001446	Syringaresinol-4'-O-glucopyranosid	Lignans and Coumarins	0.158	5.914
MA10107783	3-[(1-Carboxyvinyl)oxy]benzoic acid	Phenolic acids	0.057	3.821
Zmgn001448	2-Propylmalic Acid	Organic acids	0.047	4.615
pmb3101	2-Isopropylmalic Acid	Organic acids	0.042	5.660
pmb2928	Gallic acid-4-O-glucoside	Phenolic acids	0.0003	6907.519
pme2529	1,5-Anhydro-D-glucitol	Saccharides and Alcohols	0.0003	5491.852
mws0906	Coniferin	Phenolic acids	0.00007	11505.222
**LBB-12/LBB-0 down and LBB-60/LBB-12 down**				
pme1383	Pyridoxine	Others	0.497	0.104
pme3882	2'-Deoxyuridine	Nucleotides and derivatives	0.482	0.294
pmb3045	Tricin-7-O-Glucuronide	Flavonoids	0.415	0.186
Lmtn004049	Abscisic acid	Organic acids	0.332	0.115
Hmcp002207	Isorhamnetin-7-O-glucoside (Brassicin)*	Flavonoids	0.328	0.289
pmp000583	Diosmetin-7-O-glucuronide	Flavonoids	0.320	0.087
pmb0608	Chrysoeriol-7-O-(6''-malonyl)glucoside	Flavonoids	0.256	0.327
pma0149	Sinapoyl malate	Phenolic acids	0.250	0.433
pmp000588	Diosmetin-7-O-(6''-malonyl)glucoside	Flavonoids	0.234	0.290
mws0183	3,4-Dihydroxybenzoic acid (Protocatechuic acid)*	Phenolic acids	0.213	0.349
mws0639	2,3-Dihydroxybenzoic Acid*	Phenolic acids	0.211	0.308
mws0180	2,5-Dihydroxybenzoic acid; Gentisic Acid	Phenolic acids	0.192	0.381
mws0064	Eriodictyol (5,7,3',4'-Tetrahydroxyflavanone)	Flavonoids	0.181	0.276
mws0052	Baicalin	Flavonoids	0.178	0.048
MWSHY0031	Apigenin-7-O-glucuronide	Flavonoids	0.164	0.041
pmb0587	Chrysoeriol-7-O-(2''-O-glucuronyl)glucoside	Flavonoids	0.160	0.0004
mws1094	Aromadendrin (Dihydrokaempferol)	Flavonoids	0.158	0.002
MWSHY0121	Luteolin 7-O-glucuronide	Flavonoids	0.144	0.057

MWSHY0035	Scutellarein-7-O-glucuronide (Scutellarin)	Flavonoids	0.144	0.051
HJAP064	Isorhamnetin-3-O-(6"-malonylglucoside)	Flavonoids	0.134	0.187
pmn001702	Tetahydroxyflavone-7-O-glucuronide	Flavonoids	0.119	0.058
pme2596	4-Pyridoxic acid	Others	0.117	0.0004
Lmzn001894	Kaempferol-3-O-glucuronide	Flavonoids	0.115	0.054
Hmgn004139	Isohydroxymatairesinol	Lignans and Coumarins	0.059	0.353
Hmln002189	Quercetin-3-O-(6"-malonyl)galactoside	Flavonoids	0.029	0.103
pmb0557	Cyanidin-3-O-(3",6"-O-dimalonyl)glucoside	Flavonoids	0.024	0.105
pmb0554	Pelargonidin-3-O-(6"-O-malonyl)glucoside	Flavonoids	0.017	0.00008
pmb0542	Cyanidin-3-O-(6"-O-malonyl)glucoside	Flavonoids	0.017	0.084
pmb0562	Pelargonidin-3-O-(3",6"-O-dimalonylglucoside)	Flavonoids	0.015	0.00005
Lmcp003751	"""Cyanidin-3-O-(6""""-O-acetyl-2""""-O-xylosyl)glucoside"""	Flavonoids	0.015	0.00009
pmb0550	Cyanidin-3-O-glucoside (Kuromanin)	Flavonoids	0.011	0.225
